# β-Arrestin1 Mediates the Endocytosis and Functions of Macrophage Migration Inhibitory Factor

**DOI:** 10.1371/journal.pone.0016428

**Published:** 2011-01-25

**Authors:** Lishi Xie, Xiaohang Qiao, Yanfang Wu, Jie Tang

**Affiliations:** 1 Center for Infection and Immunity, Institute of Biophysics, Chinese Academy of Sciences, Beijing, People's Republic of China; 2 Graduate University, Chinese Academy of Sciences, Beijing, People's Republic of China; Cornell University, United States of America

## Abstract

Macrophage migration inhibitory factor (MIF) is a pleiotropic cytokine, regulating inflammatory and immune responses. MIF binds to cell surface receptor CD74, resulting in both rapid and sustained ERK activation. It was reported that MIF-induced rapid ERK activation requires its co-receptor CD44. But the exact mechanism underlying sustained ERK activation is not well understood. In the current study, we described a detailed mechanism of MIF mediated sustained ERK activation. We found that β-arrestin1, a scaffold protein involved in the activation of the MAPK cascade, interacts with CD74 upon MIF stimulation, resulting in CD74-mediated MIF endocytosis in a chlorpromazine (CPZ)-sensitive manner. β-arrestin1 is also involved in endocytotic MIF signaling, leading to sustained ERK activation. Therefore β-arrestin1 plays a central role in coupling MIF endocytosis to sustained ERK activation.

## Introduction

Macrophage migration inhibitory factor (MIF) is a ubiquitously expressed pleiotropic cytokine that functions as a pro-inflammatory mediator. MIF is involved in the pathogenesis of many inflammatory diseases and cancer development [1]. The molecular mechanism of MIF's action appears to be unique among proinflammatory cytokines. MIF induces a rapid and transient ERK activation (lasts less than 90 minutes) [2], as well as a sustained ERK activation (lasts up to 24 hours) [3]. It was reported that MIF-induced rapid ERK activation is mediated by CD74 and CD44 receptor complex. CD74 is responsible for MIF cell surface binding, and CD44 is necessary for MIF signal transduction [4]. However, the molecular mechanism underlying the sustained ERK activation induced by MIF is not clear yet. Besides CD44 and CD74, MIF has another two cell surface receptors, chemokine receptor CXCR2 and CXCR4, which are involved in MIF-mediated migratory function [5]. Although it has been reported that MIF can be taken up by both immune and non-immune cells in a temperature and energy dependent manner [6], [7], the detailed mechanism and function of the endocytosis of MIF remain unclear.

β-arrestin is a versatile adaptor well known for its role in G protein-coupled receptor (GPCR) desensitization, internalization and signal transduction [8]. New evidences indicated that β-arrestin is also a signaling molecule in single transmembrane receptor pathways, such as the IGF-1 receptor and TGF-β receptor signal pathways [9]. One of the well characterized β-arrestin signaling pathways is β-arrestin-dependent activation of the ERK/MAPK pathway. β-arrestin works as a scaffold protein to recruit Raf, MEK and ERK to the receptor, enhancing activation of ERK [10]. This protein complex remains attached to the receptor as it travels to the early endosome, thus promoting sustained MAPK signaling [11]. Given the similarity between β-arrestin and MIF in endocytosis and sustained ERK phosphorylation, we hypothesize that β-arrestin may also play a role in MIF endocytosis and subsequent signaling events.

In the current study, we demonstrated that MIF undergoes a chlorpromazine (CPZ)-sensitive endocytosis in the mouse macrophage cell line RAW264.7, and it is CD74-dependent. Upon MIF stimulation, β-arrestin1 interacts with the endocytic CD74. As an adaptor, β-arrestin1 recruits downstream signal molecules, resulting in sustained ERK activation and cell cycle progress. Therefore we described a detailed mechanism linking CD74-mediated MIF endocytosis with sustained ERK activation and defined a central role of β-arrestin in this process.

## Results

### Cellular uptake of MIF by chlorpromazine-sensitive endocytosis

**Figure 1:**
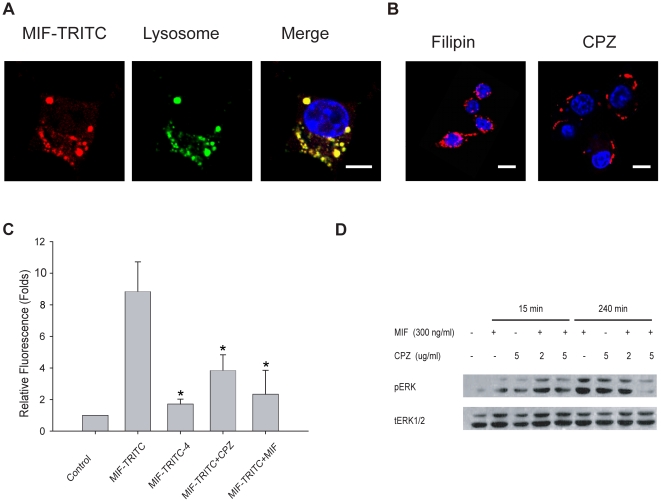
Uptake of MIF via chlorpromazine (CPZ)-sensitive endocytosis. **A**, Uptake of MIF in RAW264.7 cells. RAW264.7 cells were incubated with MIF-TRITC for 1 h at 37°C, and then stained with LysoTraker Green and Hoechst. Subcellular distribution of MIF-TRITC was imaged with confocal microscopy. **B**, Uptake of MIF-TRITC is clathrin-dependent. RAWRAW264.7 cells were pretreated with Filipin (5 µM) or CPZ (5 µg/ml) for 30 min before stimulation with MIF-TRITC for another 2 h. Staining and confocal microscopy was carried out as in **A**. **C**, Uptake of MIF-TRITC is specific and temperature dependent. RAWRAW264.7 cells were treated with MIF-TRITC under the conditions indicated. MIF endocytosis was measured by FACS and the geometric mean of fluorescent signals was calculated by normalizing the signal of each sample with the signal of untreated cells. The experiment was repeated three times and means ± S.D. are indicated. *: Statistical analysis was carried out using the Student's t-test and P<0.05 was considered statistically significant. **D**, The sustained ERK activation induced by MIF is endocytosis-dependent. Serum-starved RAW264.7 cells were treated with CPZ for 30 min before stimulation with MIF for 15 min or 240 min. Western blot analysis was carried out using anti-ERK and anti-pERK antibodies. Scale bars: 10 µm.

Previous biochemical studies have shown that the uptake of MIF is temperature and energy dependent [7], but the precise mechanism of MIF internalization remains unclear. RAW264.7 was incubated with tetramethyl rhodamine labeled biologically active MIF (MIF-TRITC) to visualize the internalization of MIF by using confocal laser scanning microscopy. At 4°C, which is a condition nonpermissive for internalization, MIF-TRITC was only detected on the cell surface (Fig. S1A). Subsequently cells were washed extensively to remove non-specific binding MIF and recovered at 37°C for a few minutes. MIF-TRITC could be observed in small endocytic vesicles, which merged with late endosomes/lysosomes afterwards (Fig. 1A). Increased intracellular accumulation of MIF-TRITC containing endocytic vesicles was observed with longer incubation time (Fig. S1A).

Endocytosis in vertebrate cells occurs by two main mechanisms: clathrin-dependent and clathrin-independent endocytosis. The latter is also known as lipid-raft-dependent endocytosis [12]. Clathrin-dependent endocytosis can be blocked by chlorpromazine (CPZ), which leads to adaptor protein complex 2 (AP2) and clathrins redistributed away from the plasma membrane [13]. On the other hand, most clathrin-independent endocytosis can be inhibited by sequestering cellular cholesterol with Filipin [14]. To investigate the mechanism of MIF endocytosis, RAW264.7 cells were pre-treated with CPZ or Filipin for 30 minutes, and then incubated with MIF-TRITC for 2 hours at 37°C. Blocking the clathrin-independent pathway by Filipin had no effect on the endocytosis of MIF-TRITC. However, inhibition of the clathrin-dependent pathway by CPZ abolished the endocytosis of MIF-TRITC and arrested MIF-TRITC at the cell surface (Fig. 1B).

Same results of the temperature and clathrin dependent endocytosis of MIF-TRITC were also observed by flow cytometry, a semi-quantitative analysis method. At 4°C, the endocytosis of MIF-TRITC was much lower than that at 37°C (Fig. 1C). Furthermore the endocytosis of MIF-TRITC could be antagonized by 10-fold excess of unlabeled MIF, suggesting that this process is receptor dependent. Finally, the endocytosis of MIF-TRITC can be efficiently suppressed by clathrin-dependent endocytosis inhibitor, CPZ (Fig. 1C).

### The endocytosis of MIF contributes to sustained ERK activation

Endocytosis and cell-surface receptor recycling are crucial for the magnitude, duration and nature of signaling cascade [12]. To assess whether MIF endocytosis is associated with ERK activation, a representative signaling event upon MIF stimulation [3], we investigated the rapid and sustained ERK phosphorylation after MIF stimulation. In RAW264.7 cells, ERK activation was detectable 10 min after MIF treatment and lasted more than 12 hours. The peak of ERK activation appeared from 60 to 240 min, which is consistent with the time frame of MIF endocytosis (Fig. S1B). In addition, sustained ERK activation (240 min) induced by MIF can be attenuated by CPZ pretreatment. In contrast, the rapid ERK activation (15 min) induced by MIF was not inhibited, but rather was slightly elevated (Fig. 1D). This elevated rapid ERK activation may be explained by longer sequestration of MIF on the cell surface after CPZ treatment.

### CD74 is involved in the endocytosis of MIF

The likely involvement of clathrin in the endocytosis of MIF and the association of endocytosis with sustained ERK activation prompted us to investigate the role of MIF receptors in this process. The first identified MIF cell surface receptor, CD74, is a type II transmembrane receptor that constantly recycles between the cell surface and the cytosol [6]. Recent studies have found that depletion of clathrin or AP-2 results in increased CD74 expression on the cell surface [15]. In addition, as the cell surface receptor of MIF, CD74 contributes to MIF-induced ERK activation [6].

**Figure 2:**
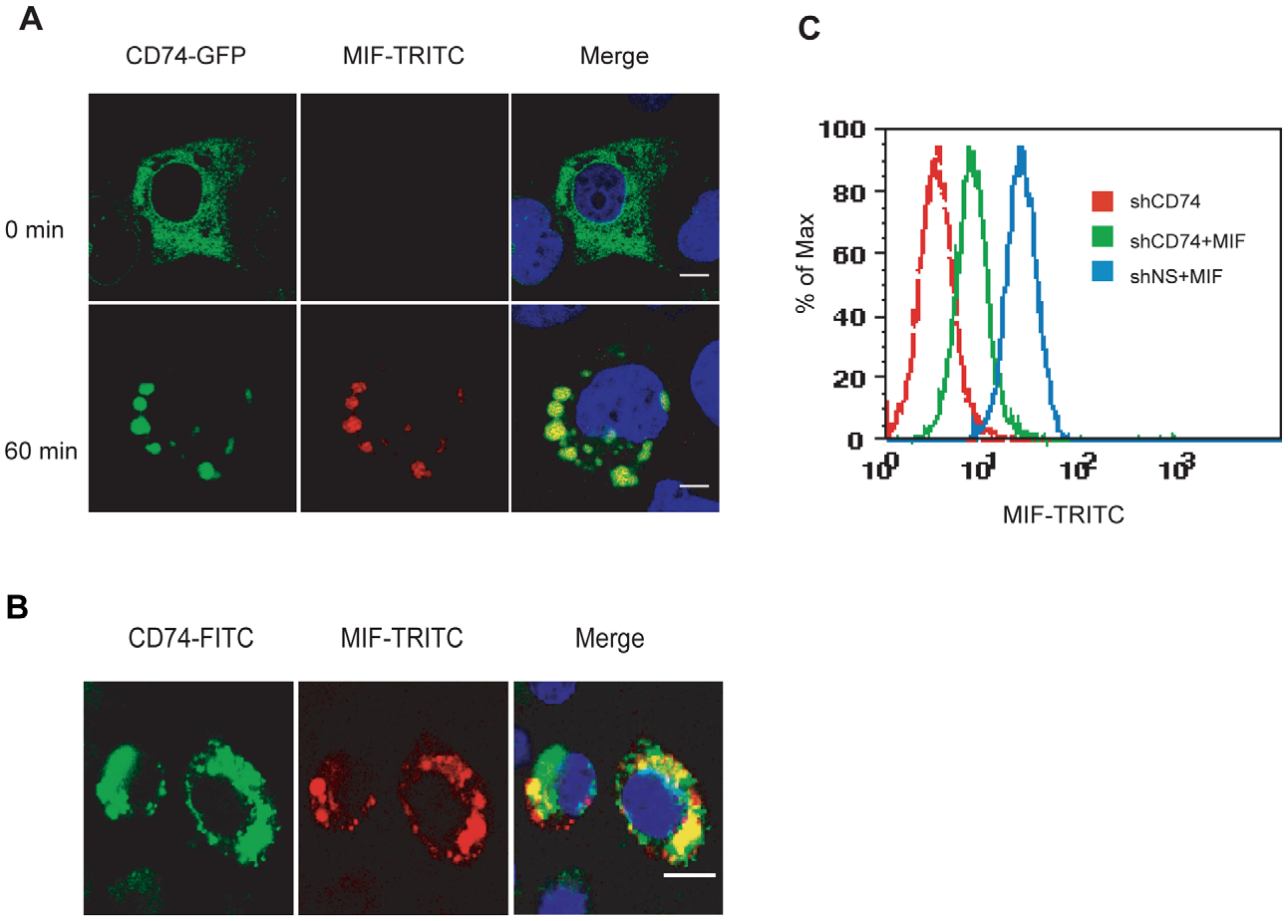
CD74 contributes to MIF endocytosis. **A**, CD74 co-localizes with internalized MIF in COS-7 cells. COS-7 cells were transiently transfected with GFP-CD74, and stimulated with (bottom) or without (upper) MIF-TRITC for 1 h. MIF-TRITC and GFP-CD74 localization was imaged with confocal microscopy. **B**, Internalized MIF-TRITC co-localizes with endogenous CD74. Serum-starved RAW264.7 cells were stimulated with MIF-TRITC for 1 h. Permeablized cells were labeled with anti-CD74-FITC antibody and imaged with confocal microscopy. **C**, MIF endocytosis is CD74-dependent. CD74 specific shRNA (shCD74) or non-specific shRNA (shNS) stably transfected RAW264.7 cells were incubated with MIF-TRITC and analyzed with FACS. Scale bars: 10 µm.

To test whether CD74 plays a role in MIF endocytosis, we used an N-terminal GFP-fused CD74 to follow its dynamic within the cell after MIF stimulation. GFP-CD74 or GFP plasmid was transiently transfected into COS-7 cells which are CD74 deficient [6] and MIF-TRITC was added to the cells 36 hours after transfection. While MIF cannot be taken up by GFP transfected cells (data not shown), it can be internalized by GFP-CD74 transfected cells. Before MIF stimulation, GFP-CD74 was evenly distributed in the cytosol and cell surface. However, GFP-CD74 became co-localized with MIF-TRITC in endosomes after 60 min's incubation (Fig. 2A). Similar results were observed in RAW264.7 cells (Fig. S2A), suggesting that MIF was internalized via a CD74-mediated pathway.

Using similar strategy, we investigated whether other MIF receptors were involved in the endocytosis of MIF. CD44-GFP and CXCR4-GFP did not co-localize with MIF-TRITC in RAW264.7 cells upon stimulation, whereas CXCR2-GFP partially co-localized with MIF-TRITC (Fig. S2B). Moreover, the endogenous CD74 was also found co-localized with MIF-TRITC in RAW264.7 cells by immunofluorescence (Fig. 2B). But the endogenous staining of CD44 didn't show co-localization with MIF-TRITC (data not shown). To confirm that CD74 is required for MIF endocytosis, we knocked down CD74 expression in RAW264.7 cells by RNA interference (Fig. S3A). Flow cytometry results showed that MIF-TRITC uptake was significantly reduced as a result of CD74 depletion (Fig. 2C). Thus, CD74 is required for MIF endocytosis.

### β-arrestin1 is required for the endocytosis of MIF and subsequent sustained ERK activation

β-arrestin exists as two isoforms, β-arrestin1 and β-arrestin2, both of which are important in the regulation of GPCR desensitization, internalization and signaling events. Recent evidences suggested that β-arrestin can also regulate the endocytosis and signaling of non-GPCR receptors [9].

**Figure 3:**
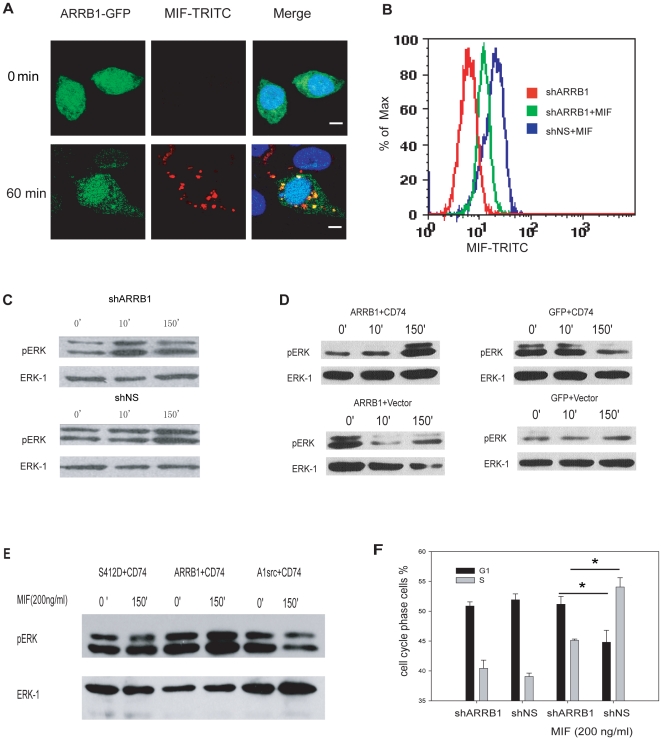
β-arrestin1 is involved in MIF endocytosis and sustained ERK activation. **A**, β-arrestin1 is co-localized with MIF in RAW264.7 cells. RAW264.7 cells expressing ARRB1-GFP were either unstimulated (top) or stimulated (bottom) with MIF-TRITC for 1 h and subsequently fixed. Cellular distribution of β-arrestin1 and MIF-TRITC was imaged by confocal microscopy. Scale bars: 5 µm **B**, MIF endocytosis is β-arrestin1-dependent. β-arrestin1 knocked-down RAW264.7 cells (shARRB1) were stimulated with MIF-TRITC and analyzed with FACS. **C–E**, β-arrestin1 is involved in sustained ERK activation induced by MIF. **C**, RAW264.7 cells stably transfected with a β-arrestin1-specific (shARRB1) and non-specific shRNA (shNS) plasmid were stimulated with MIF (200 ng/ml) for 10 min or 150 min. Cell extracts were subjected to Western blotting with anti-pERK and anti-ERK antibodies. **D,E** Same as **C**, except COS-7 cells were transiently co-transfected with plasmids as indicated. Serum-starved cells were stimulated with MIF (200 ng/ml) for 10 min or 150 min, and **F**, β-arrestin1 is important for the cell cycle progress induced by MIF. RAW264.7 cells stably transfected with shARRB1 or shNS were stimulated with MIF (200 ng/ml) for 12 h and stained with propidium iodide. The samples were then subjected to FACS analysis and the percentages of cells in the G1 and S phases were calculated. The experiment was repeated three times and the results are shown as means ± S.D. * Statistical analysis was carried out using the Student's t-test and P<0.05 was considered statistically significant. ARRB1, β-arrestin1; S412D, β-arrestin1 mutant; A1Src, β-arrestin mutant.

To test whether β-arrestin is involved in the endocytosis of MIF, we followed the localization of β-arrestin before and after MIF-TRITC incubation. In stable β-arrestin1-GFP expressing RAW264.7 cells, β-arrestin1-GFP evenly distributed in the cytosol. Upon MIF-TRITC stimulation β-arrestin-GFP accumulated into some vesicles and co-localized with MIF-TRITC bearing vesicles (Fig. 3A). Similar results were observed in COS-7 cells. β-arrestin1-GFP or β-arrestin2-GFP constructs were transiently co-transfected with CD74-myc in COS-7 cells and MIF-TRITC uptake was examined 36 hours after transfection. Only β-arrestin1-GFP and MIF-TRITC were found co-localized in endosomes after stimulation (Fig. S4A). To confirm that β-arrestin1 contributes to MIF endocytosis, we knocked down β-arrestin1 in RAW264.7 cells by RNA interference (Fig. S3B). MIF-TRITC uptake was significantly reduced as a result of β-arrestin1 depletion (Fig. 3B).

As a scaffold protein for endocytosis and signaling in GPCR, β-arrestin associates with specific components of the MAPK cascade [10]. To investigate the role of β-arrestin1 in MIF-induced ERK activation, we tested the ability of MIF to induce ERK activation after β-arrestin1 depletion in RAW264.7 cells. MIF-induced sustained ERK activation was impaired in β-arrestin1 shRNA transfected RAW264.7 cells compared to non-specific shRNA transfected cells. However, there was no significant difference in rapid ERK activation upon MIF treatment (Fig. 3C).

COS-7 cells express very low level of endogenous β-arrestin [16], and MIF-induced sustained ERK activation was not observed in COS-7 cells, even with CD74 over-expression [4]. To test whether β-arrestin1 can rescue MIF-induced sustained ERK activation in COS-7 cells, we co-expressed β-arrestin1 and CD74 transiently in COS-7 cells. Upon MIF stimulation, sustained ERK activation was observed in co-transfected cells, but not in cells expressing only β-arrestin1 or CD74 (Fig. 3D). To investigate in more detail how β-arrestin1 affects sustained ERK activation after MIF stimulation, two β-arrestin1 mutants were introduced. β-arrestin1 S412D mutant cannot target receptors to clathrin-coated pits, thus disturbs β-arrestin1 mediated receptor endocytosis. β-arrestin1 (P91G-P121E) mutant (A1src), with the c-Src binding site disrupted, is defective in ERK activation, but not in β-arrestin1-mediated endocytosis [17]. MIF-induced sustained ERK activation was not observed when S412D or A1src mutant was co-expressed with CD74 in COS-7 cells (Fig. 3E). Our results suggest that β-arrestin1 serves as an important adaptor for MIF endocytosis and subsequent signal transduction by providing a scaffold for clathrin, c-Src and components of MAPK cascade.

Sustained ERK activation is required for cell cycle progression through G1 phase [18]. Inhibition of ERK activation causes cells to be arrested at the G1 phase [19]. In addition, it was reported that sustained ERK activation and related cell cycle progression in the IGF-1 receptor pathway is mediated by β-arrestin1 [9]. Since we have demonstrated that β-arrestin1 is involved in MIF-initiated sustained ERK activation, we further investigated the role of β-arrestin1 in MIF-induced cell cycle progression. As shown in Fig. 3F, MIF induced cell cycle progression was inhibited by β-arrestin1 knockdown in RAW264.7 cells, compared to mock cells, whilst in non-stimulated cells, β-arrestin1 knockdown did not affect the cell cycle.

### β-arrestin1 binds to CD74 and links MIF endocytosis to sustained ERK activation

β-arrestin bridges ERK activation with the endocytosis of the corresponding receptors. β-arrestin recruits downstream signaling molecules to the clathrin-coated pits that contain receptors. Along with these signaling complexes, β-arrestins internalizes with receptor containing endocytic vesicles to prolong the signal activation [20]. We observed that β-arrestin1 co-localized with internalized MIF-TRITC, and CD74 was also present in MIF containing vesicles in COS-7 and RAW264.7 cells. It suggests that β-arrestin1 may link MIF endocytosis to sustained ERK activation by interacting with CD74 directly.

**Figure 4:**
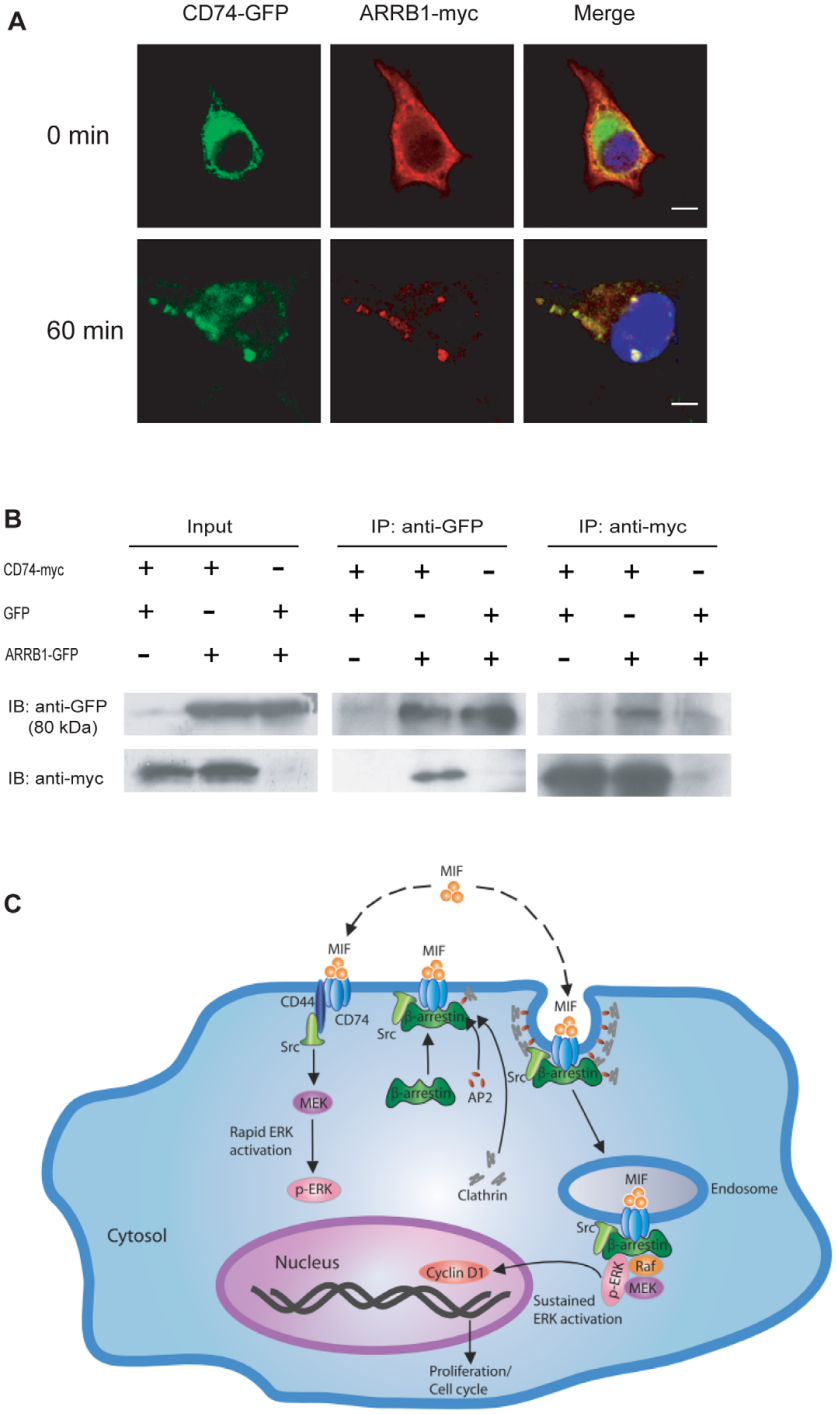
β-arrestin1 interacts with CD74 upon MIF stimulation. **A**, CD74 co-localizes with β-arrestin1 upon MIF stimulation. GFP-CD74 and ARRB1-myc-expressing COS-7 cells were stimulated with MIF (200 ng/ml) for 1 h, and stained with anti-myc antibody and TRITC-labeled secondary antibody. The cellular distribution of β-arrestin1 and CD74 was imaged by confocal microscopy. Scale bars: 10 µm **B**, β-arrestin1 interacts with CD74. COS-7 cells were co-transfected with the plasmids indicated and stimulated with MIF (200 ng/ml) for 1 h. Cell lysates were immunoprecipitated with the antibodies indicated. Whole cell lysates (Input) and immunoprecipitates (IP) were assayed by western blot with the antibodies indicated. **C**, Model for β-arrestin1 mediated sustained ERK activation induced by MIF endocytosis. Upon MIF stimulation, β-arrestin1 translocates to MIF-CD74 endosomes and binds to CD74, serving as a scaffold for recruiting downstream signal molecules, such as Raf, MEK and pERK. The sustained ERK activation stabilized by β-arrestin1 is required for Cyclin D expression and cell cycle progression.

Results from confocal microscopy showed that upon MIF stimulation β-arrestin1 translocated from cytosol to plasma membrane and later ended up in the same vesicles along with CD74 (Fig. 4A). Further investigation showed that β-arrestin1 interacts with CD74 in reciprocal co-immunoprecipitation experiments (Fig. 4B), and this interaction is MIF-dependent (Fig. S4B).

## Discussion

The results presented here suggested a novel mechanism underlining MIF induced sustained ERK activation. Upon MIF stimulation, β-arrestin1 binds to CD74, triggers MIF and CD74 complex internalization, and serves as a scaffold for recruiting downstream signal molecules, such as components of the MAPK/ERK cascade. Subsequently, MIF endocytic vesicles entry into cytosol along with the β-arrestin1 scaffolds. By stabilizing Raf/MEK/ERK complex, β-arrestin1 maintains MIF-induced ERK activation for a comparatively long period of time (Fig. 4C).

MIF was one of the first cytokines discovered [21]. An important feature of MIF signaling is its ability to induce both rapid and sustained ERK activation [2], [3]. Via this pathway MIF regulates cell proliferation [3] and adhesion-dependent signaling responses [22]. MIF signaling is known to induce a rapid ERK activation through a receptor complex comprising CD74 and CD44 [4], which is depends on Jab1, a intracellular binding partner of MIF, and Src kinase activity[2].The sustained ERK activation induced by MIF was suggested to be mediated by autocrine MIF, and Rho GTPase, MLCK activation may be involved in this process [3], [23], while Jab1 inhibits the sustained ERK activation likely by blocking MIF secretion[2], [24]. However, since intracellular MIF also binds to and activates MLCK [25] and extracellular MIF is efficiently endocytosed and translocated into the cytosol, it is possible that the autocrine action of extracellular MIF in sustained ERK signalling involves MIF endocytosis. We found that reduction of MIF uptake was followed by attenuation of sustained ERK activation after CPZ pretreatment, which suggested the association and consecution between MIF endocytosis and its sustained ERK activation.

Previous biochemical studies have shown that the uptake of MIF is temperature and energy dependent [7], presented confocal and flow cytometry results showed that it was a CPZ sensitive process, these evidences suggests that the endocytosis of MIF may be a clathrin-dependent pathway, while because of the limitation of the chemical inhibitors, exact pathway cannot be determined at the present time.It was reported that MIF-induced sustained ERK activation was proposed to occur through a receptor-mediated signaling pathway [2]. Our confocal microscopy results showed that the endocytosed MIF co-localized with ectopic expressed CD74 and endogenous CD74. Depletion of CD74 not only repressed the endocytosis of MIF, but also inhibited MIF-induced sustained ERK activation. These all suggested that the endocytosis of MIF and downstream sustained ERK activation are both mediated by receptor, CD74. We also studied the roles of other three cell surface receptors of MIF in this process. The endocytosed MIF didn't co-localize with GFP-tagged CD44 and CXCR4, which were ruled out firstly. Correspondingly, endocytosed MIF partially co-localized with GFP-tagged CXCR2. It was recently reported that CD74 interacts with CXCR2 [5] or CXCR4 leading to alternative signaling pathways such as Akt activation [26]. Those signal transduction pathways may share similar mechanism that using β-arrestins as mediators [27]. Those alternative pathways may be the subject for future studies.

β-arrestin1 is an adaptor protein for GPCR, as well as non-GPCR, and it can mediate receptor internalization and signaling to the mitogen-activate protein kinases ERK, JNK, and p38 as well as Akt, PI3K, and RhoA [8]. In this study, we found that β-arrestin1 interacted with CD74 upon MIF stimulation and played a central role in coupling MIF endocytosis with downstream signaling. Src phosphorylation is a necessary step in the activation of various mitogenic signaling pathways including the MAPK/ERK pathway. It has been reported that c-Src is required for MIF induced rapid ERK activation [2], [4], but the Src binding site in the intracellular domain of CD74 is not clear. Here we found that MIF dependent sustained ERK activation is significantly attenuated when β-arrestin1 mutant A1src (deficient in c-Src binding) was introduced. Thus our work also explained how Src recruitment by β-arrestin1 connects with MIF-induced sustained ERK activation.

MIF plays important role in maintenance of steady state Cyclin D1 expression and cell proliferation in immortalized rodent fibroblasts through a number of signaling elements, including ERK activation [23]. Importantly, Cyclin D1 expression is not activated by transient ERK signalling but is only triggered after sustained activation of this pathway [28], [29], [30]. Our data links CD74-mediated MIF endocytosis and downstream signal transduction in the context of cell cycle progression. It has been reported that MIF expression is correlated with cancer prognosis, specifically for hepatocellular carcinomas, colon cancers and prostate cancers [31], [32], [33]. MIF may indirectly facilitate tumor growth via promotion of angiogenic response [34], [35]. Our result also suggests a new way by which MIF may contribute to tumorigenesis: facilitating cell cycle progression and proliferation through β-arrestin1 mediated sustained ERK activation. The linkage between ERK activation and cell cycle progression induced by MIF can also shed light on inflammatory diseases in terms of persistence of inflammatory cells or growth of intrinsic cells. It has been reported that antigen-induced arthritis is reduced in MIF deficient mice, which is associated with reduction of splenocyte proliferation and T cell activation dependent on ERK activation [36].

This is the first study that defines a role for β-arrestin1 in the endocytosis of MIF and the downstream signaling events, providing an important link in the biology of MIF. Cellular signal transduction involves highly coordinated cascades of events. Our results showed that MIF employs β-arrestin1 as a molecular scaffold to maintain integrity and specificity of signaling. Understanding molecular mechanism of MIF-induced sustained ERK activation is imperative for both basic research and practical applications, and it will help us to find new ways to treat related diseases by fine tuning of this pathway.

## Materials and Methods

### Cell Culture

COS-7 and RAW264.7 cells (both from ATCC) were cultured in Dulbecco's modified Eagle's medium plus 10% (v/v) heat-inactivated fetal bovine serum. All cell culture was carried out at 37°C in a humidified incubator with 5% CO_2_. Plasmids were transiently transfected into the cells using Lipofectamine 2000 (Invitrogen). Plasmid constructs and sequences of oligo used are listed in the supporting information.

### Antibodies and reagents

p-ERK, ERK-1, Myc, tubulin and actin specific antibodies were purchased from Santa Cruz Biotechnology. ARRB1/2 (A1CT) antibody was kindly provided by Dr. Lefkowitz. Chlorpromazine (CPZ) and the nuclear dye Hoechst 33342 were purchased from Sigma-Aldrich. The lysosome dye, LysoTracker® Green DND-26 (L7526) was purchased from Molecular Probes™.

### Plasmid constructs

The pCI-EGFP plasmid (GFP fused to the C terminus of the targeted protein) was generated by inserting the EGFP coding sequence into Sal I and Not I sites of the pCI-neo vector (Promega). ARRB1-GFP and GFP-CD74 were constructed by subcloning a full-length human β-arrestin1 cDNA and CD74 cDNA into the EcoRI and Sal I sites of pCI-EGFP. Myc-tagged ARRB1 and CD74 were generated by subcloning a full-length β-arrestin1 cDNA and a CD74 cDNA fragment into the pCI-neo vector containing the coding sequence of the Myc epitope. The β-arrestin1 mutant A1src was kindly provided by Dr. Lefkowitz. shRNAs against murine β-arrestin1 and CD74 was obtained by cloning the target sequence into pSUPER (Oligoengine Inc., Seattle, WA, USA) RNAi vector. The oligonucleotides used in this study are indicated in Supplementary Information (Table S1).

### MIF stimulation

Recombinant human MIF was purified from an expression system as previously described [37] and contaminating endotoxin is inactivated by Polymyxin B [38]. MIF is labeled with TRITC (Sigma-Aldrich) as described before [39]. For ERK activation studies, RAW264.7 cells were plated at a density of 4×10^5^ cells/ml and allowed to grow for 12 h in high glucose DMEM with 10% FBS. The cells were starved in DMEM with 0.1% (v/v) FBS for 12 h. Without a medium change, the cells were treated with 5 µg/ml CPZ (Sigma-Aldrich) for 30 min, recombinant MIF was added at 200 ng/ml unless otherwise specified. For endocytosis assay by flow cytometry, RAW264.7 cells were serum-starved for 2 h before each endocytosis assay. Cells were incubated with 1 µg/ml MIF-TRITC for the indicated time at 37°C, washed once with citric acid buffer (132 mM citric acid and 66 mM NaH_2_PO_4_), three times with PBS, and then fixed with 4% paraformaldehyde and subjected to flow cytometry analysis (FACS Calibur™; Becton Dickinson).

### Western Blotting

Whole cell extracts were prepared from cells after the indicated treatments. Cells first were washed with cold PBS, and then ice-cold RIPA buffer (containing 1 mM Na_3_VO_4_, 1 mM NaF, and a protease inhibitor mixture (Roche)) was added. After incubation on ice for 30 min and centrifugation at 12,000 rpm for 15 min (4°C), the supernatant was removed, and the protein concentration was determined. Equal amounts of cellular proteins were fractionated on SDS-PAGE gels and transferred to PVDF membranes (Millipore). Immunoblotting was performed with indicated antibodies.

### Co-immunoprecipitation

Cells were lysed with immunoprecipitation buffer (10 mM Tris–HCl, pH 7.4, 150 mM NaCl, 1 mM EDTA, 1 mM EGTA, 0.2 mM Na_3_VO_4_, 1% Triton X-100 plus 0.5% NP-40) containing protease inhibitors. 500 µl of cell lysate was incubated with 2 µg of appropriate antibodies for 30 min at 4°C. 15 µl of Protein A/G bead slurry (Santa Cruz) was added for another 60 min. Immunoprecipitates were separated by SDS-PAGE electrophoresis and then blotted with the antibodies indicated.

### Cell cycle analysis

Cells were fixed in 70% ethanol for 24 h. After removing the ethanol by washing with PBS, incubate cells with 100 µg/ml RNase A (DNase-free; Sigma), and 50 µg/ml propidium iodide. At least 1×10^4^ cells from each sample were analyzed for DNA content using a BD Calibur flow cytometer. Percentages of cells in G1, S, and G2/M phases were determined using ModFIT LT software (Verity Software House).

### Immunofluorescence

COS-7 expressing GFP-CD74 and ARRB1-myc were serum starved, washed with PBS, fixed with 4% paraformaldehyde, permeabilized in 0.1% Triton X-100/PBS, and then blocked with 5% bovine serum albumin in PBS for 1 h. Myc antibody was used to probe for transient β-arrestin1 expression in COS-7 for 1 h at room temperature. Cells were washed with PBS, and incubated with PE-conjugated rabbit secondary antibody for 1 h at RT, washed, then mounted with Vectashield. Immunofluorescence images were obtained using Olympus FV500 laser scanning confocal microscope.

## Supporting Information

Table S1 **Oligonucleotides Used in This Study.**
(DOC)

Figure S1 **Time course of MIF uptake and induced ERK activation in RAW 264.7.** (A) Uptake of MIF-TRITC in RAW 264.7 cells. Cells were pre-incubated with MIF-TRITC (1 µg/ml) at 4°C for 30 min, then washed extensively and were incubated at 37°C for the times indicated. Cells were stained with LysoTraker Green and Hoechst. (B) The time course of MIF-induced ERK activation. Serum-starved RAW 264.7 cells were stimulated with MIF (200 ng/ml) for the times indicated. The cell lysates were separated with SDS-PAGE electrophoresis and blotted with ERK and phospho-ERK antibodies. CPZ: chlorpromazine, MIF-TRITC: tetramethyl rhodamine-labeled MIF.
(TIF)

Figure S2 **CD74 but not other receptors contributes to MIF endocytosis.** (A) CD74 co-localizes with endocytic MIF in RAW 264.7 cells. GFP-CD74 stably expressed RAW 264.7 cells were stimulated with (bottom) or without (upper) MIF-TRITC (1 µg/ml) for 60 min and imaged with confocal microscopy. (B) Co-localization of endocytic MIF with its receptor, CD44, CXCR2 and CXCR4. RAW264.7 cells were transiently transfected either with CXCR2-GFP, CXCR4-GFP or CD44-GFP plasmid and serum-deprived overnight before being stimulated with 1 µg/ml of MIF-TRITC for 60 min. Cellular distribution of MIF-TRITC and its receptors was imaged by confocal microscopy. Scale bars: 5 µm.
(TIF)

Figure S3 **Stable knockdown of CD74 and β-arrestin1 in RAW 264.7 cells.** (A) RAW 264.7 cells were stably tranfected with CD74 and a non-specific shRNA interference plasmid. Whole cell extracts were subjected to Western blotting with CD74 and β-tubulin antibodies. (B) RNA interference successfully knocked down endogenous β-arrestin1. RAW 264.7 cells were stably transfected with a β-arrestin1-specific or non-specific RNA interference plasmid. Cell extracts were separated by SDS-PAGE electrophoresis and subjected to Western blotting with β-arrestin1/2 and β-tubulin-specific antibodies.
(TIF)

Figure S4 **β-arrestin1 interacts with CD74 upon MIF stimulation.** (A) β-arrestin1 is co-localized with MIF in COS-7 cells when CD74 present. COS-7 cells expressing ARRB1-GFP and CD74-myc were either unstimulated (top) or stimulated (bottom) with MIF-TRITC (1 µg/ml) for 60 min and subsequently fixed. Cellular distribution of β-arrestin1 and MIF-TRITC was imaged by confocal microscopy. (B) Interaction of CD74 with β-arrestin1 is dependent on MIF stimulation. COS-7 cells were transiently co-transfected with ARRB1-GFP and CD74-myc plasmids for 36 h. Transfected cells were stimulated with or without MIF (200 ng/ml) for 1 h. The cell extracts were immunoprecipitated and blotted with the antibodies indicated. Cell lysates were immunoprecipitated with the antibodies indicated. Immunoblots with the antibodies indicated were used to analyze whole cell lysates (Input) and immunoprecipitates (IP). ARRB1, β-arrestin1.
(TIF)
