# Supplementary material for: β-Arrestin1 Mediates the Endocytosis and Functions of Macrophage Migration Inhibitory Factor
Source: PLoS One. 2011 Jan 25;6(1):e16428. doi: 10.1371/journal.pone.0016428 (PMC3026819; doi:10.1371/journal.pone.0016428)
Supplement: Table S1 — Oligonucleotides Used in This Study. (DOC) [file pone.0016428.s001.doc]

| **Table S1** |  |
| --- | --- |
|  |  |
| Name | Squence（5’->3’） |
| Primers |  |
| GFP-F-SalI | TCTAGAGTCGACATGGTGAGCAAGGGCGAGGAGCTGTTC |
| GFP-R-Flag | ATCGTCATCGTCCTTGTAATCCTTGTACAGCTCGTCCATGCCGAGAGTG |
| Flag-R-s-NotI | AGGGAAGCGGCCGCTCACTTATCGTCATCGTCCTTGTAATC |
| hCD74-F-EcoRI | GGCTAGCGAATTCATGCACAGGAGGAGAAGCAGGAG |
| hCD74-R-SalI | CACCATGTCGACCATGGGGACTGGGCCCAGATCCTGCTT |
| hArrt1-F-SalI | CACCATGTCGACCGGACCATGGGCGACAAAGGGA |
| hArrt1-R-MluI | AGTATTCACGCGTCTGTTGTTGAGCTGTGGAGAGCCGGTA |
| hArrt1S412D-MluI | AGTATTCACGCGTCTGTTGTTGAGCTGTGGATCGCCGGTA |
| hArrt2-F-XbaI | GGTACCTCTAGATCTACCATGGGGGAGAAACCCGGGACCAGGGTCTT |
| hArrt2-R-SalI | CACCATGTCGACGCAGAGTTGATCATCATAGTCGTCATCCTT |
| mArrt1-F-EcoI | ACAGACGAATTCCATGGGCGACAAAGGGACAC |
| mArrt1-R-SalI | TCTATCGTCGACTCTGTTGTTGAGGTGCGGAGAGC |
| shRNA |  |
| shNS | GCGCGCTTTGTAGGATTCC |
| shmARRB1-Target1 | GGCCTGTGGTGTGGATTAT |
| shmARRB1-Target2 | AGCCTTCTGTGCTGAGAAC |
| shmCD74-Target | CGTCCAATGTCCATGGATA |
