# Supplementary figures and images for: β-Arrestin1 Mediates the Endocytosis and Functions of Macrophage Migration Inhibitory Factor
Source: PLoS One. 2011 Jan 25;6(1):e16428. doi: 10.1371/journal.pone.0016428 (PMC3026819; doi:10.1371/journal.pone.0016428)

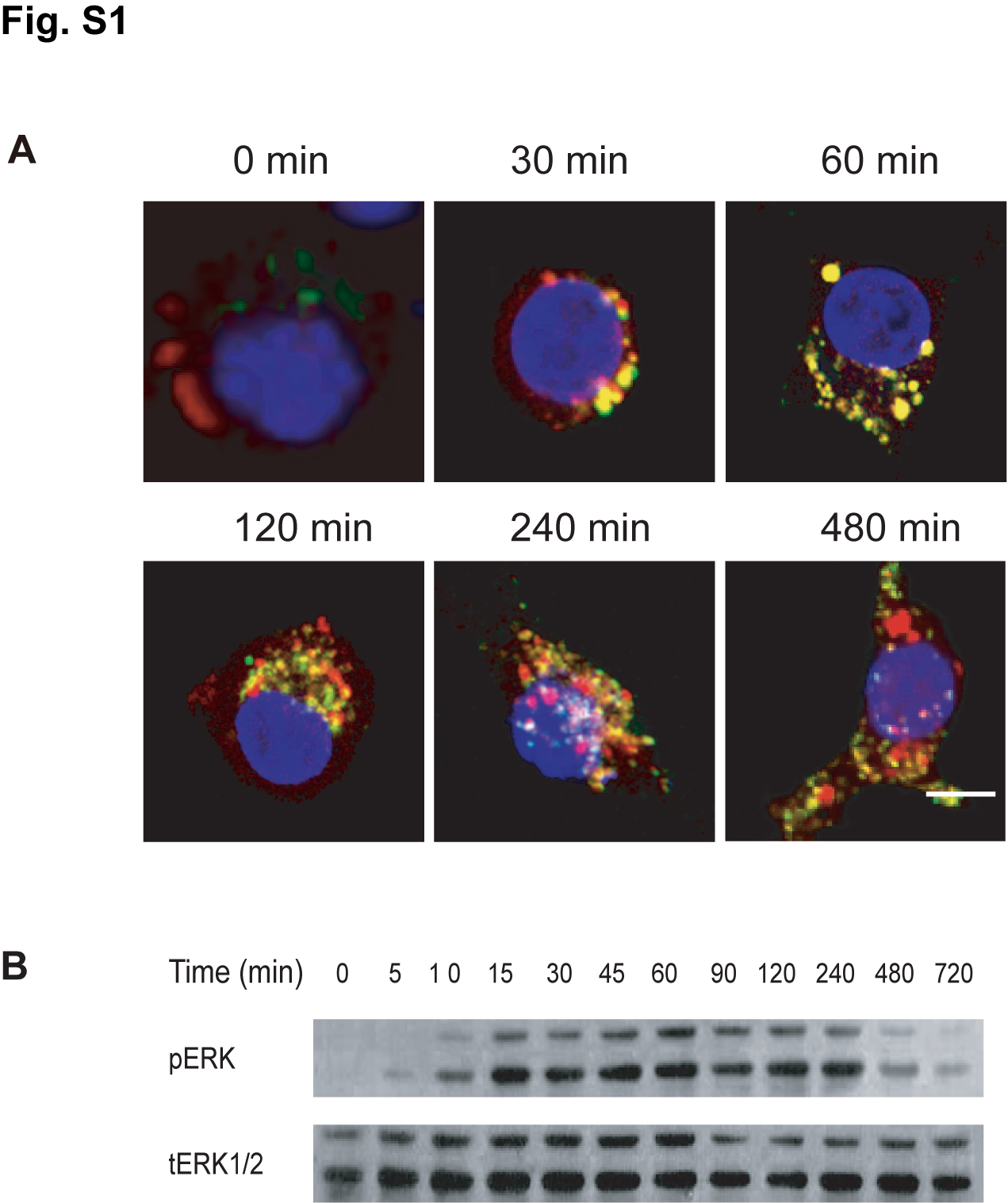

Supplement: Figure S1 — Time course of MIF uptake and induced ERK activation in RAW 264.7. (A) Uptake of MIF-TRITC in RAW 264.7 cells. Cells were pre-incubated with MIF-TRITC (1 µg/ml) at 4°C for 30 min, then washed extensively and were incubated at 37°C for the times indicated. Cells were stained with LysoTraker Green and Hoechst. (B) The time course of MIF-induced ERK activation. Serum-starved RAW 264.7 cells were stimulated with MIF (200 ng/ml) for the times indicated. The cell lysates were separated with SDS-PAGE electrophoresis and blotted with ERK and phospho-ERK antibodies. CPZ: chlorpromazine, MIF-TRITC: tetramethyl rhodamine-labeled MIF. (TIF) [file pone.0016428.s002.tif]

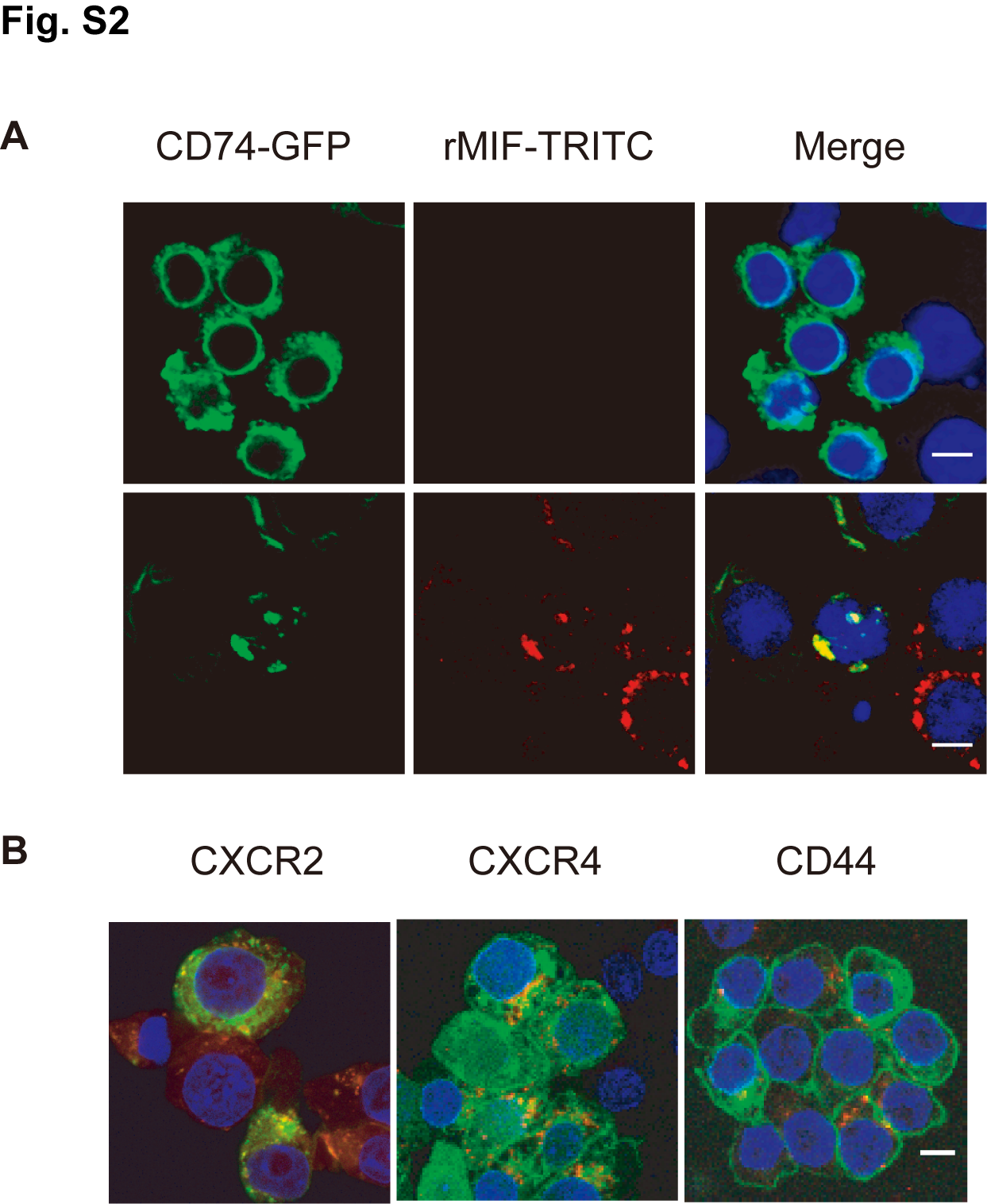

Supplement: Figure S2 — CD74 but not other receptors contributes to MIF endocytosis. (A) CD74 co-localizes with endocytic MIF in RAW 264.7 cells. GFP-CD74 stably expressed RAW 264.7 cells were stimulated with (bottom) or without (upper) MIF-TRITC (1 µg/ml) for 60 min and imaged with confocal microscopy. (B) Co-localization of endocytic MIF with its receptor, CD44, CXCR2 and CXCR4. RAW264.7 cells were transiently transfected either with CXCR2-GFP, CXCR4-GFP or CD44-GFP plasmid and serum-deprived overnight before being stimulated with 1 µg/ml of MIF-TRITC for 60 min. Cellular distribution of MIF-TRITC and its receptors was imaged by confocal microscopy. Scale bars: 5 µm. (TIF) [file pone.0016428.s003.tif]

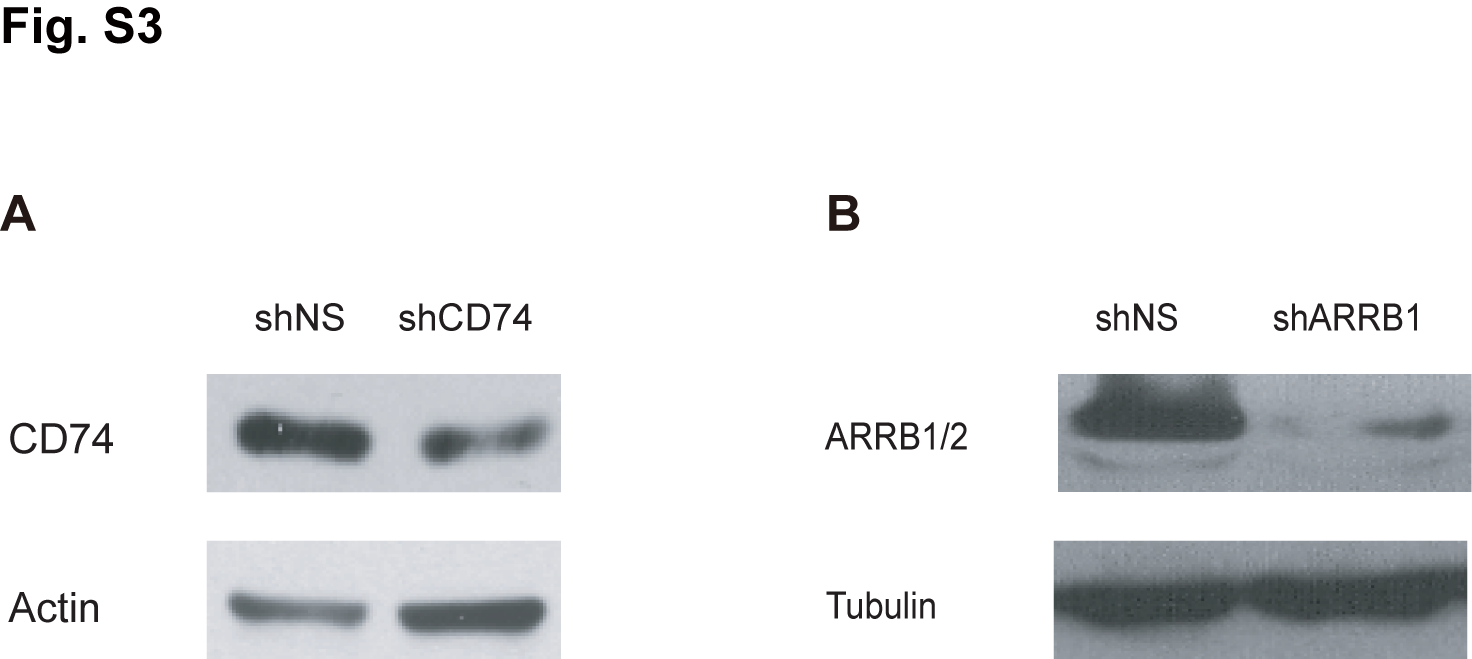

Supplement: Figure S3 — Stable knockdown of CD74 and β-arrestin1 in RAW 264.7 cells. (A) RAW 264.7 cells were stably tranfected with CD74 and a non-specific shRNA interference plasmid. Whole cell extracts were subjected to Western blotting with CD74 and β-tubulin antibodies. (B) RNA interference successfully knocked down endogenous β-arrestin1. RAW 264.7 cells were stably transfected with a β-arrestin1-specific or non-specific RNA interference plasmid. Cell extracts were separated by SDS-PAGE electrophoresis and subjected to Western blotting with β-arrestin1/2 and β-tubulin-specific antibodies. (TIF) [file pone.0016428.s004.tif]

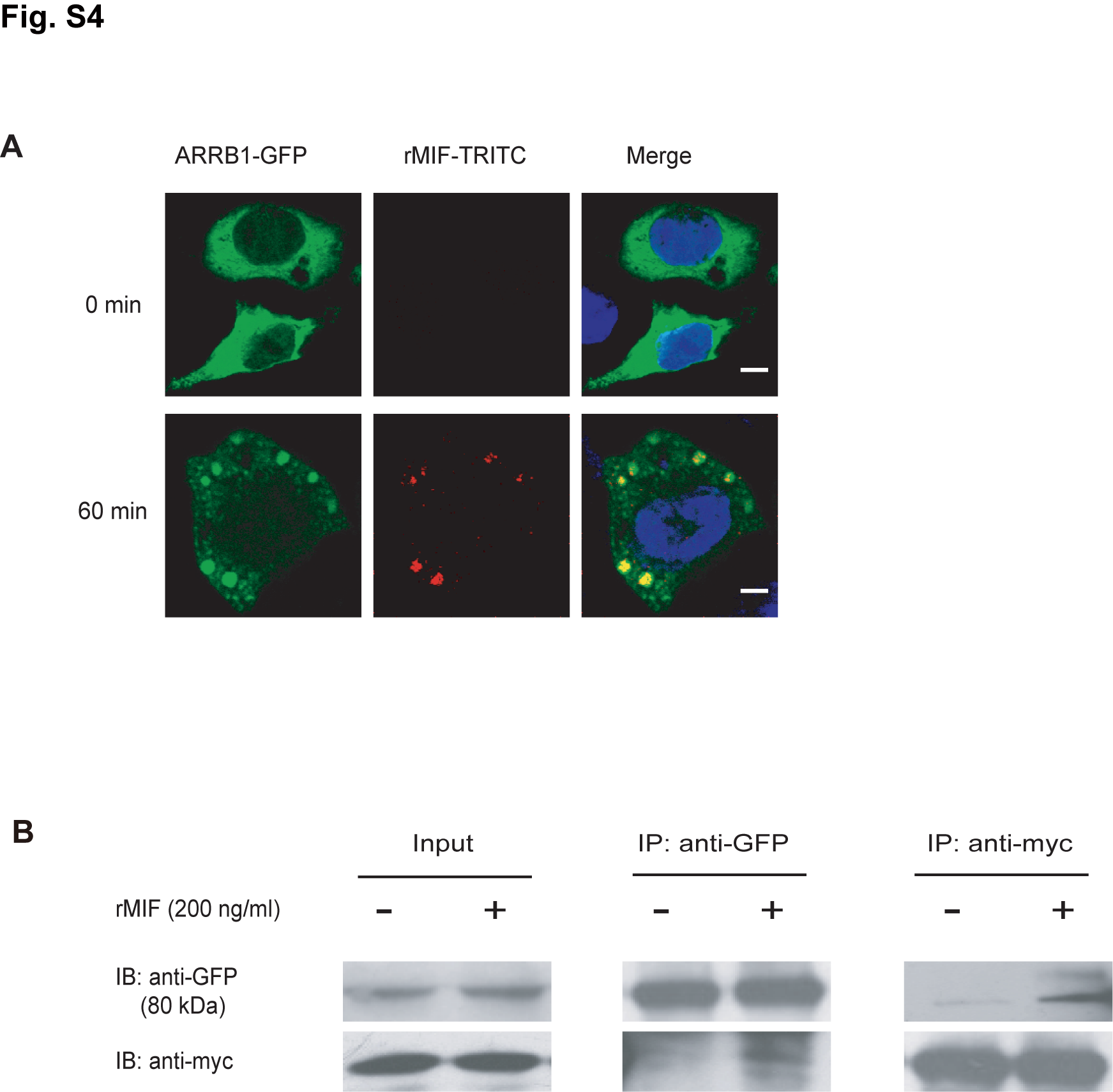

Supplement: Figure S4 — β-arrestin1 interacts with CD74 upon MIF stimulation. (A) β-arrestin1 is co-localized with MIF in COS-7 cells when CD74 present. COS-7 cells expressing ARRB1-GFP and CD74-myc were either unstimulated (top) or stimulated (bottom) with MIF-TRITC (1 µg/ml) for 60 min and subsequently fixed. Cellular distribution of β-arrestin1 and MIF-TRITC was imaged by confocal microscopy. (B) Interaction of CD74 with β-arrestin1 is dependent on MIF stimulation. COS-7 cells were transiently co-transfected with ARRB1-GFP and CD74-myc plasmids for 36 h. Transfected cells were stimulated with or without MIF (200 ng/ml) for 1 h. The cell extracts were immunoprecipitated and blotted with the antibodies indicated. Cell lysates were immunoprecipitated with the antibodies indicated. Immunoblots with the antibodies indicated were used to analyze whole cell lysates (Input) and immunoprecipitates (IP). ARRB1, β-arrestin1. (TIF) [file pone.0016428.s005.tif]
